# Single-cell analysis reveals differential regulation of the alveolar macrophage actin cytoskeleton by surfactant proteins A1 and A2: implications of sex and aging

**DOI:** 10.1186/s13293-016-0071-0

**Published:** 2016-03-18

**Authors:** Nikolaos Tsotakos, David S. Phelps, Christopher M. Yengo, Vernon M. Chinchilli, Joanna Floros

**Affiliations:** Center for Host Defense, Inflammation and Lung Disease (CHILD) Research, Department of Pediatrics, The Pennsylvania State University College of Medicine, Rm. C4752, H085, 500 University Drive, PO Box 850, Hershey, PA 17033-0850 USA; Department of Cellular and Molecular Physiology, The Pennsylvania State University College of Medicine, Hershey, PA USA; Department of Public Health Sciences, The Pennsylvania State University College of Medicine, Hershey, PA USA; Department of Obstetrics and Gynecology, The Pennsylvania State University College of Medicine, Hershey, PA USA

**Keywords:** Surfactant protein A, Alveolar macrophages, Actin cytoskeleton, F-actin, G-actin, Single-cell imaging analysis, Sex differences, Age-related differences, Immune response

## Abstract

**Background:**

Surfactant protein A (SP-A) contributes to lung immunity by regulating inflammation and responses to microorganisms invading the lung. The huge genetic variability of SP-A in humans implies that this protein is highly important in tightly regulating the lung immune response. Proteomic studies have demonstrated that there are differential responses of the macrophages to SP-A1 and SP-A2 and that there are sex differences implicated in these responses.

**Methods:**

Purified SP-A variants were used for administration to alveolar macrophages from SP-A knockout (KO) mice for in vitro studies, and alveolar macrophages from humanized SP-A transgenic mice were isolated for ex vivo studies. The actin cytoskeleton was examined by fluorescence and confocal microscopy, and the macrophages were categorized according to the distribution of polymerized actin.

**Results:**

In accordance with previous data, we report that there are sex differences in the response of alveolar macrophages to SP-A1 and SP-A2. The cell size and F-actin content of the alveolar macrophages are sex- and age-dependent. Importantly, there are different subpopulations of cells with differential distribution of polymerized actin. In vitro, SP-A2 destabilizes actin in female, but not male, mice, and the same tendency is observed by SP-A1 in cells from male mice. Similarly, there are differences in the distribution of AM subpopulations isolated from SP-A transgenic mice depending on sex and age.

**Conclusions:**

There are marked sex- and age-related differences in the alveolar macrophage phenotype as illustrated by F-actin staining between SP-A1 and SP-A2. Importantly, the phenotypic switch caused by the different SP-A variants is subtle, and pertains to the frequency of the observed subpopulations, demonstrating the need for single-cell analysis approaches. The differential responses of alveolar macrophages to SP-A1 and SP-A2 highlight the importance of genotype in immune regulation and the susceptibility to lung disease and the need for development of individualized treatment options.

## Background

Surfactant protein A (SP-A) is one of the many molecules that contribute to lung immunity. It binds foreign particles and organisms that invade the lungs and targets them for clearance via phagocytosis by the alveolar macrophages. It also enhances clearance of inflammatory cells after the inflammation has been resolved and finally gets removed by the alveolar macrophages themselves. The SP-A/alveolar macrophage interaction enhances the alveolar macrophage functions such as chemotaxis, chemokine and reactive oxidant production, phagocytosis, and endolysosomal trafficking.

SP-A knockout mice have extensively been used as a model to elucidate the role of SP-A in lung innate immunity. It has been shown that SP-A regulates inflammation following ozone exposure [1] and other lung challenges [2], while its absence leads to increased susceptibility to infection by many organisms, such as *Klebsiella pneumoniae* [3, 4], *Pseudomonas aeruginosa* [5], group B *Streptococcus* [6], and viruses [7]. Humans, however, have two distinct SP-A genes, namely, SP-A1 and SP-A2, and a number of variants for each one, indicating that the roles of SP-A in immunity are complex and finely tuned. The different SP-A variant molecules display distinct functions, including, but not limited to, cytokine production [8, 9], phosphatidylcholine secretion [10], and phagocytic activity [11–13].

Previous studies performed in our laboratory showed that the proteome of alveolar macrophages from SP-A knockout (KO) mice treated with a single intrapharyngeal dose of SP-A resembles that of the wild-type mice [14] and there are sex differences in the response of the alveolar macrophage proteome to SP-A [15]. The regulation of the alveolar macrophage phenotype by SP-A becomes more complex when one takes into account the fact that the proteomes of alveolar macrophages derived from humanized transgenic mice that express either SP-A1 or SP-A2 are significantly different [16] and that there are sex differences in the responses of the macrophage cellular proteomes to different SP-A variants [17].

The alveolar macrophages perform most of the search-and-destroy functions (chemotaxis and phagocytosis) in the lung. The actin cytoskeleton is a crucial mediator of these processes. Interestingly, the proteomic analyses mentioned above identified proteins that are related to the actin cytoskeleton as being differentially regulated by SP-A1 and SP-A2. In the present study, we performed a single-cell imaging analysis to determine the in vitro and ex vivo effects of SP-A1 and SP-A2 on the distribution of F-actin in the alveolar macrophages. Our findings demonstrate diverse roles of SP-A1 and SP-A2 in the regulation of the alveolar macrophage cytoskeleton and, hence, the cell’s motility and activation status.

## Methods

### Animals

All mice used in the present study were on the C57BL6/J background and were either 8 weeks (young) or 8–10 months (old) in age. SP-A KO and humanized transgenic SP-A1, SP-A2, and SP-A1/SP-A2 mice were generated on the C57BL6/J background [18]. The animals were raised in the breeding facility of the Penn State College of Medicine. All the mice were maintained in a pathogen-free environment or in barrier facilities with free access to food and water. The study was approved by the Institutional Animal Care and Use Committee of the Penn State College of Medicine. For each experiment, equal numbers of age-matched males and females (*n* = 3) were used.

### Collection of bronchoalveolar lavage fluid

Mice were euthanized using a mixture of ketamine and xylazine, and bronchoalveolar lavage (BAL) fluid was collected [15] by instilling 1 mM EDTA/PBS into the lungs through a tracheal cannula using 0.5 mL of solution five times, for a total of 2.5 mL. For each instillation, the solution was applied and withdrawn three times with concurrent chest massage. The BAL fluid was centrifuged at 150*g* for 5 min at 4 °C, and the cell pellet was washed once with 1 mL of 1 mM EDTA/PBS. Total cells were counted with the use of a hemocytometer, and cytocentrifuge slides were prepared for differential cell counting with the Fisher Healthcare Protocol Hema 3 stain (Fisher Scientific) according to the manufacturer’s instructions.

### Culture of mAMs

Following collection of the BAL fluid, AMs were washed with serum-free RPMI-1640 containing 2 mM glutamine and 1× antibiotic-antimycotic solution. The AMs were then resuspended in the same medium and plated on UV-sterilized coverslips (No. 1, 18-mm diameter) in 12-well cell culture plates. After allowing the cells to attach for 90 min, the medium was changed and the cells were incubated overnight at 37 °C in the presence of 5 % CO_2_. In a set of experiments, the AMs were treated with 10 μg of SP-A1, 10 μg of SP-A2, 5 μg SP-A1 + 5 μg SP-A2, or 10 μg SP-A1 + 10 μg SP-A2 for 60 min before staining.

### Preparation of purified SP-A

Purified SP-A was prepared from CHO cells as described previously [8]. Briefly, stably transfected CHO-derived cell lines expressing either SP-A1 (6A^2^) or SP-A2 (1A^0^) were cultured for 5 days in the expression medium as described [8], and the conditioned media were collected. SP-A was purified using mannose affinity chromatography, concentrated, and stored at −80 °C until use. The concentration of lipopolysaccharides (LPS) in the SP-A preparations was measured using the Limulus Amebocyte Lysate QCL-1000 assay (Lonza, Walkersville, MA). LPS was below the detection limit of the assay in all the preparations used in the present study. Purity of SP-A was determined by silver staining (Bio-Rad Silver Stain Plus Kit, Bio-Rad).

### Staining for F-actin, G-actin, and cell membranes

Following the overnight culture, the AMs were washed once with PBS, fixed with 3.7 % paraformaldehyde for 10 min at room temperature, permeabilized with 0.5 % Triton X-100, and washed three times before incubation for 30 min in staining solution, containing one unit of Alexa Fluor 488-conjugated phalloidin (Molecular Probes, Eugene, OR). In some experiments, the staining solution also contained 0.3 μM of Alexa Fluor 594-conjugated (deoxyribonuclease 1) DNase I and 5 μg/mL of Alexa Fluor 647-conjugated wheat germ agglutinin (Molecular Probes). Following three more washes, the coverslips were mounted on cover glasses with ProLong Gold Mounting Medium with DAPI (Life Technologies, Eugene, OR). Depending on the distribution of F-actin, the cells were blindly (without knowledge of the sex or the genotype of the animal) categorized as belonging to one of the four subpopulations: A, minimal F-actin staining; B, perinuclear F-actin staining; C, diffuse cytoplasmic F-actin; or D, existence of cytoplasmic protrusions (filopodia or podosomes).

### Image acquisition and data analysis

For light microscopy experiments of F-actin staining, the mAMs were imaged using a Nikon TE-2000 PFS fluorescence microscope, using a ×60/1.40 phase contrast, oil immersion objective lens. The images were captured using a Photometrics Coolsnap HQ2 digital camera (0.11 μm/pixel) and saved as TIFF files. The acquisition time was 100 ms for all images acquired. Nikon NIS-Elements v.3.0 software was used for image acquisition, and Adobe Photoshop CD4 was used for image analysis. The AMs were analyzed by manually drawing an area around the border of each cell, and a cell-free area of equal size was used for background subtraction. The exported data included the average fluorescence per pixel, the number of pixels in each selected area (cell), and the sum of fluorescence intensity for each cell.

For confocal microscopy experiments (multicolor imaging), a Leica AOBS SP8 laser scanning confocal imaging microscope (Leica, Heidelberg, Germany) at the Penn State College of Medicine Imaging Core was used. Images were acquired using a high-resolution Leica ×60/1.3 Plan-Apochromat oil immersion objective lens. The laser lines were produced by a UV diode (for DAPI) and an 80-MHz white light laser (Leica SP8 AOBS module, for Alexa Fluor conjugates). The emission signals were collected sequentially using acousto-optical beamsplitter (AOBS) tunable filters using a pinhole Airy size of 1.0. The bandwidths of the highly sensitive HyD detectors were set in a way that prevented fluorescence bleed-through. The images were obtained with the use of the Leica Application Suite (LAS AF), and image analysis was performed using the Imaris v.7.3 software (Bitplane). The fluorescent signals were rendered with the Surface tool in the Surpass View of the software, and the statistics from each channel were exported to Microsoft Excel spreadsheets.

### Statistical analysis

All statistical analyses were performed with GraphPad Prism v.6.0 and SAS v.9.4. Data are displayed as the mean ± SEM. Comparisons of means were analyzed with one-way ANOVA or two-tailed unpaired *t* test with Welch’s correction for non-equal variances. In certain experiments, two-way ANOVA was used for sex (male vs. female) and genotype (KO vs. SP-A1 vs. SP-A2), followed by planned comparisons using Fisher’s least significant difference. Comparisons of frequencies were performed with chi-square contingency analysis tests. In order to account for interindividual differences among the animals used in the study, a hierarchical analysis (animal–culture well–cell) was designed in the SAS software consisting of generalized linear mixed-effects models with Poisson regression. The models contain random effects to account for (1) the correlation due to measurements from the same animal, and for (2) similar environmental conditions within a well, followed by comparisons of genotype (or treatment, in the case of in vitro experiments) and phenotype, along with Bonferroni corrections for multiple comparisons. A *P* value ≤0.05 was considered statistically significant.

## Results

### Cell area and F-actin content in the alveolar macrophages of humanized transgenic SP-A mice

**Fig. 1:**
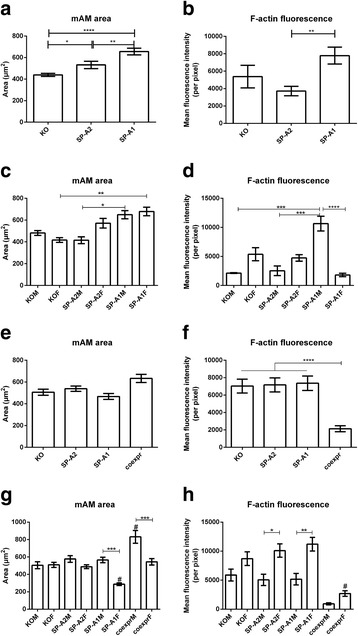
Cell area and mean F-actin fluorescence per pixel of alveolar macrophages of the specified genotypes. **a**–**d** 8–10-month-old mice. **e**–**h** 2-month-old mice. All data shown represent measurements made on cells obtained from three mice (*n* = 3). Panels **c**, **d** and **g**, **h** represent the data from panels **a**, **b** and **e**, **f**, respectively, taking into account the sex of the animals. Number of cells per bar ranges from *n* = 22 to *n* = 79. Comparisons were made by two-way ANOVA followed by Fisher’s LSD test. *P ≤ 0.05; **P ≤ 0.01; ***P ≤ 0.001; ****P ≤ 0.0001; #significant difference from all other genotypes of the same sex

Following previous studies that highlighted the importance of SP-A in the macrophage cytoskeleton [14, 17], we measured the cell size and F-actin content of alveolar macrophages isolated from young and old transgenic mice carrying either the SP-A1 (6A^2^) or SP-A2 (1A^0^) gene. We examined both the cell area and the mean per pixel fluorescence intensity of phalloidin staining as a measurement of the F-actin content per cell [14]. The macrophages of old mice (8–10 months old) that carry either the SFTPA1 (SP-A1) or the SFTPA2 (SP-A2) cDNA were significantly larger than the macrophages from the KO mice (Fig. 1a). In fact, the macrophages from the SP-A1 mice had a significantly larger area than the ones from the SP-A2 mice. Upon examination of the mean F-actin fluorescence per pixel, the macrophages from the SP-A1 mice showed significantly higher fluorescence intensity compared to those from both KO and SP-A2 (Fig. 1b). Taken together, these data indicate that there are higher levels of polymerized actin in the alveolar macrophages from SP-A1 mice than in the ones from SP-A2 or KO mice.

In order to determine whether there are sex differences among the macrophages from different genetic backgrounds, we analyzed the same data taking into account the sex of the animals. Two-way ANOVA indicated that there is no sex-by-genotype interaction affecting the area of the macrophages (*F*(2,12) = 0.26, *P* = 0.7733). Sex was not found to be a factor that influences the area of AMs in old mice (*F*(1,12) = 0.1035, *P* = 0.4738). In accordance with previously demonstrated differences in AM size in response to SP-A1 or SP-A2 proteins in vitro [14], the main effect for genotype was significant (*F*(2,12) = 1.100, *P* = 0.0057). We used post hoc tests to compare the effects of SP-A1 and SP-A2 on the area of the AMs. In both males and females, the AM area of SP-A1 mice is significantly larger compared to that of KO. In SP-A1 males, the area is significantly larger than their SP-A2 counterparts. As far as the females are concerned, the only significant difference observed is between the SP-A1 and the KO mice (Fig. 1c). Interestingly, the significant difference in cell size observed between the SP-A2 and KO genotypes (Fig. 1a) is not reflected when the analysis includes the sex of the donor animals, because male SP-A2 AMs seemingly tend to be smaller than male KO cells, but AMs from female SP-A2 tend to occupy larger area. As far as the fluorescence intensity of F-actin is concerned, the interaction effect is significant (*F*(2,12) = 2.124, *P* < 0.00001). Fluorescence intensity is significantly higher in the SP-A1 male mice compared to KO males and SP-A2 males.

In order to determine whether the SP-A-induced changes in the alveolar macrophage cytoskeleton are innate to the mice or show as a cumulative result of the prolonged exposure to SP-A, we performed the same study using young (8 weeks old) animals, either KO or expressing SP-A1, SP-A2, or both (co-expressors). When the analysis is performed without factoring in the sex of the animals, there is no significant difference in the area of the alveolar macrophages among the different groups (Fig. 1e), but the F-actin fluorescence of the co-expressors was significantly reduced in comparison to the KO, SP-A1, and SP-A2 macrophages (Fig. 1f). However, there are significant differences in both area and actin fluorescence that are masked by the exclusion of sex as a factor, as can be seen in Fig. 1g, h. Two-way ANOVA with sex and genotype as the factors demonstrates that the sex-by-genotype interaction is significant for the cell area of the AMs (*F*(3,16) = 0.6264, *P* = 0.0285). Post hoc analysis reveals that the area of the macrophages of male mice expressing both SP-A1 and SP-A2 is significantly larger than the area of KO male, SP-A2 male, and SP-A1 male mice. Among female mice, the area of the macrophages from SP-A1 mice is significantly smaller than the area from the cells from all the other female mice. When comparing mice of opposite sex, while the KO and the SP-A2 macrophages do not have sex differences, the SP-A1 and the co-expressing female macrophages have significantly smaller area than the males of the same genetic background (Fig. 1g). There is no significant effect of the sex-by-genotype interaction on the F-actin fluorescence intensity (*F*(3,16) = 0.1969, *P* = 0.4029), but a significant effect was observed for each factor (for sex, *P* = 0.0001; for genotype, *P* < 0.0001). Post hoc tests show that the F-actin fluorescence intensity is strikingly reduced in both male and female co-expressing macrophages. With the exception of the co-expressing mice, there are no differences among the different genotypes within the same sex, but there is a significant increase in the staining intensity of the cytoskeleton of cells from female mice compared to male mice of the same background (Fig. 1h). The same tendency exists in both KO and co-expressing mice, but it does not reach significant levels.

Overall, these data confirm that the actin-related cytoskeleton of alveolar macrophages is affected by different SP-A variants in a complicated way and the effects of SP-A seem to accumulate over time. Previous work has shown that in vivo administration of SP-A1 to mice influenced the actin-related proteins of AMs from male and female mice differently [17], which is in accordance with the results presented here (Fig. 1d, g, and h). These results may have functional consequences, since the ratio of SP-A1 to total SP-A in BAL has been shown to change depending on age and on whether the patients suffer from pathologic conditions, such as cystic fibrosis and alveolar proteinosis [19], and asthma [20].

### Subpopulations of alveolar macrophages based on the distribution of F-actin

**Fig. 2:**
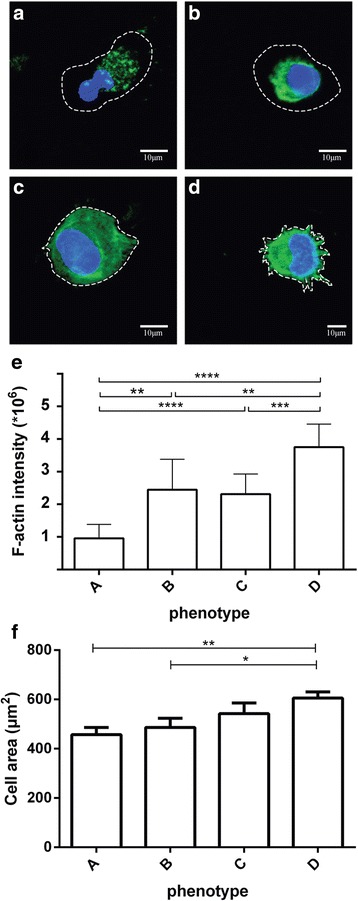
Phenotypes of the subpopulations of alveolar macrophages based on F-actin distribution. **a** Punctate stain indicates scattered cytoplasmic F-actin. **b** F-actin is found only in the juxtanuclear region. **c** F-actin is diffuse in the cytoplasm. **d** F-actin is cortical with cytoplasmic protrusions (podosomes). The *dashed lines* outline the periphery of the cells. *Scale bars* 10 μm. **e** Quantification of the F-actin content in the cells. Total number of cells, *n* = 1627. **f** Cell area of the cells for each phenotype. A subset of the cells (*n* = 305) from **e** was used for area measurements. Comparisons were made by two-tailed *t* test with Welch’s correction (equal variances not assumed). *P < 0.05; **P ≤ 0.01; ***P ≤ 0.001; ****P ≤ 0.0001

It has long been known that the alveolar macrophages are a diverse set of cells, with many subpopulations of distinct phenotypes and responses to disease or to environmental challenges [21–24]. Our initial imaging study revealed that the distribution of F-actin was not identical in all the alveolar macrophages from the bronchoalveolar fluid of mice. In order to confirm this observation, we performed confocal imaging of the phalloidin-stained alveolar macrophages. We identified four distinct phenotypes based on phalloidin staining. We named them phenotypes A, B, C, and D, in an order of increasingly activated status: (a) largely depolymerized actin, with actin “puncta” discernible throughout the cytoplasm (Fig. 2a); (b) actin tightly packed in the perinuclear region (Fig. 2b); (c) actin is diffuse in the cytoplasm (Fig. 2c); and (d) actin is taking part in the formation of cytoplasmic protrusions, i.e., filopodia and podosomes (Fig. 2d). Of note, there were cells that were negative for phalloidin staining, despite their seemingly intact nuclei. These cells were omitted from the study. All cells were blindly categorized as belonging to one of the phenotypes, cell area and phalloidin staining fluorescence were measured, and the respective measurements for each cell were backtracked to the animal of origin after the completion of each experiment. In order to verify that there are differences among the phenotypes, we compared the intensity of phalloidin staining from all cells. Phenotypes A and D are significantly different from all other phenotypes, but there are no differences in the F-actin content between phenotypes B and C (Fig. 2e). As far as the area of the cells is concerned, phenotype D is significantly different than phenotypes A and B (Fig. 2f).

### Differences in the monomeric actin pools among the alveolar macrophage subpopulations

**Fig. 3:**
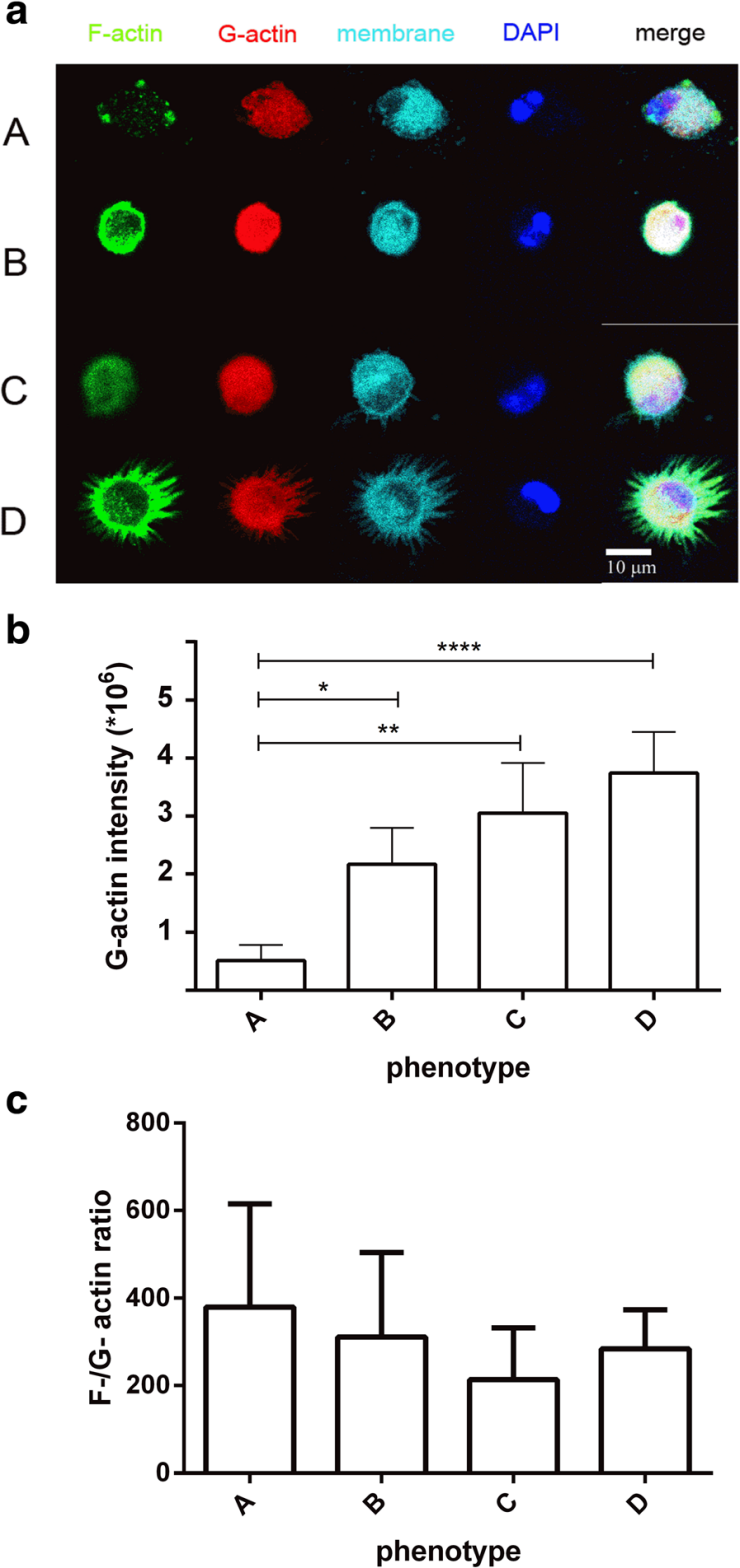
G-actin content and F-/G-actin ratio of the alveolar macrophage subpopulations. **a** G-actin and cell membrane stain of the alveolar macrophage subpopulations. Cells were fixed, permeabilized, and stained with Alexa Fluor 488-conjugated phalloidin, Alexa Fluor 594-conjugated DNase I, and Alexa Fluor 647-wheat germ agglutinin. Images were acquired sequentially with a Leica SP8 AOBS laser scanning confocal system with software-adjusted detection spectra to avoid bleed-through of the signals. The pseudocolors were assigned by ImageJ. *Scale bars* 10μΜ. **b** Means of G-actin signal intensity per phenotype. Total number of cells, *n* = 286. **c** Means of F-/G-actin ratio per phenotype. All cells from **b** were used for the measurements. Comparisons were made by two-tailed *t* test with Welch’s correction (equal variances not assumed). *P ≤ 0.05; **P ≤ 0.01; ****P ≤ 0.0001

Since the phenotypic differences among the different alveolar macrophage subpopulations based on F-actin content are so prominent, we examined whether the G-actin content shows similar differences among the phenotypes. This would give some insight as to whether the observed distinct phenotypes are a result of cytoskeleton remodeling, or a more permanent condition that affects the pools of monomeric actin as well. In a subset of the experiments, we co-stained the cells with Alexa Fluor 488-conjugated phalloidin and Alexa Fluor 594-conjugated DNase I, which has been shown to specifically bind to G-actin (monomeric) within the cell [25] (Fig. 3a). The G-actin content shows an increasing trend from phenotype “A” to phenotype “D,” with significant differences between the pairs A-B, A-C, and A-D (Fig. 3b). This result indicates that the observed phenotypes are the result of changes in gene expression that include the actin cytoskeleton, and not the result of acute events that would lead to cytoskeleton remodeling. If that were the case, the total cellular actin content (F-actin + G-actin) would not differ among the different phenotypes. Notably, there are no differences among the phenotypes in the F-/G-actin ratio (Fig. 3c), which means that the degree of F-actin polymerization does not differ among the phenotypes.

### In vitro administration of SP-A proteins alters the alveolar macrophage subpopulations

**Fig. 4:**
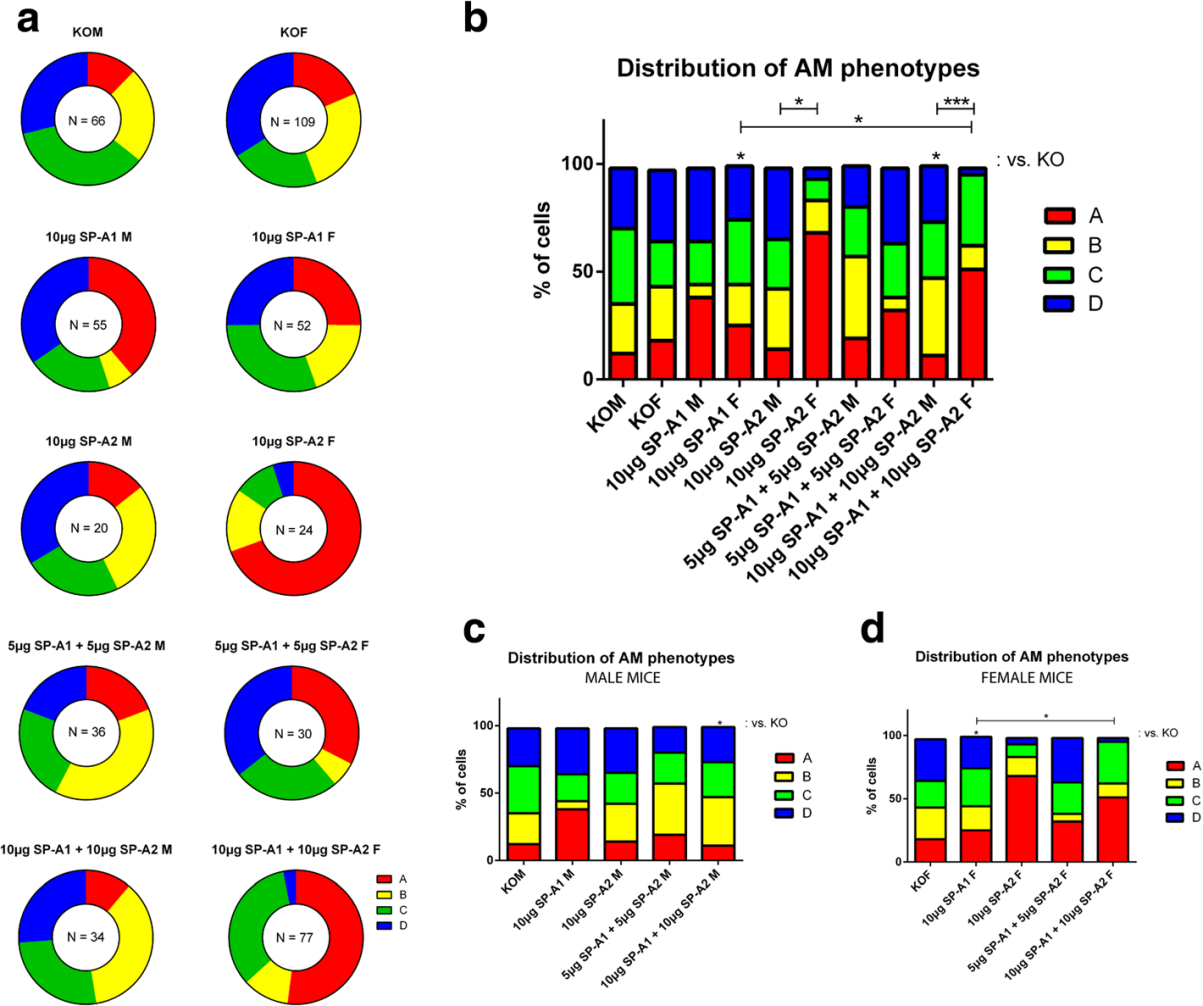
Effects of in vitro administration of SP-A1 and SP-A2 to the distribution of alveolar macrophages. **a** *Donut charts* of the distribution of the alveolar macrophage phenotypes following administration of the indicated doses of SP-A protein(s) for 60 min. The number of cells counted per genotype can be seen in the *donut hole*. **b** Statistic comparisons of data from panel **a** showing sex differences were performed in SAS with a hierarchical model accounting for cells from the same animal and cells that were cultured within the same well. **c** Data from panel **a** were replotted to show statistical comparisons of data for male mice showing differences among SP-A protein treatment regimes. **d** Data from panel **a** were replotted to show statistical comparisons of data for female mice showing differences among SP-A protein treatment regimes. *P ≤ 0.05; ***P ≤ 0.001

In order to determine whether SP-A has any effect on the frequency of the alveolar macrophage subpopulations, we examined whether in vitro short-term administration of SP-A would have any acute effects on the frequency of the phenotypes. After isolating alveolar macrophages from SP-A KO mice as described above, we added SP-A1, SP-A2 (10 μg each), or both (low dose 5 μg each or high dose 10 μg each) to the cultured macrophages for 1 h before fixing and staining the cells. While there were no baseline differences among the controls, there were significant sex differences, as a response of alveolar macrophages to SP-A2, and a combination of both SP-A1 and SP-A2 in the higher dose used, confirming that the response to surfactant proteins is, at least partially, sex-dependent and also dose-dependent (as the combination of the proteins in the lower dose did not yield significant difference) (Fig. 4a, b). In males, the distribution among the phenotypes of the cells exposed to a high dose of both SP-A1 and SP-A2 was significantly different from that of KO mice (Fig. 4c). In females (Fig. 4d), SP-A2 leads to an increase of the “A” phenotype subpopulation and a concurrent decrease of the “D” subpopulation, but this effect does not reach significance due to the small number of cells counted. SP-A1 has significant differences with both KO and the high combination dose, signifying that the two proteins may have opposing roles in the regulation of AMs. Furthermore, administration of a high dose of both proteins in females leads to a moderate, yet still significant, increase of the “A” phenotype compared to KO, which verifies the observation that SP-A1 and SP-A2 have opposing effects.

### Effects of SP-A1 and SP-A2 on the distribution of alveolar macrophage phenotypes

**Fig. 5:**
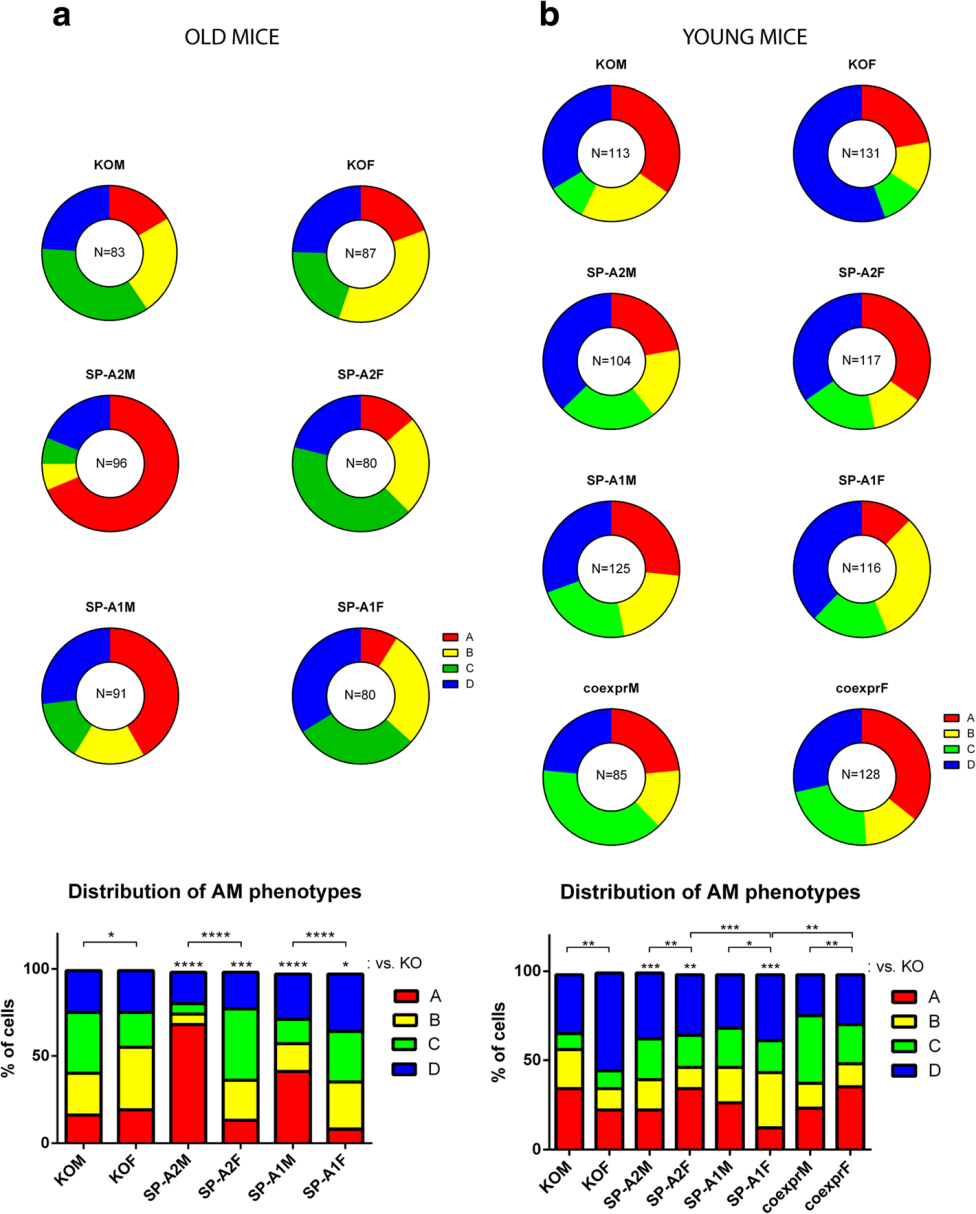
Distribution of alveolar macrophages from mice of the indicated genotypes. **a** *Donut charts* of the distribution of the alveolar macrophage phenotypes in old mice of the indicated genotype. The number of cells counted per genotype can be seen in the *donut hole*. The statistical comparisons were performed in SAS with a hierarchical model accounting for cells from the same animal and cells that were cultured within the same well and can be seen in the *stacked bar chart* at the *bottom panel*. **b** *Donut charts* of the distribution of the alveolar macrophage phenotypes in young mice of the indicated genotype. The number of cells counted per genotype can be seen in the *donut hole*. The statistical comparisons were performed pairwise as in the old mice and can be seen in the *stacked bar chart* at the *bottom panel*. *P ≤ 0.05; **P ≤ 0.01; ***P ≤ 0.001; ****P ≤ 0.0001

In order to determine whether SP-A1 and SP-A2 have similar effects on the frequency of the alveolar macrophage subpopulations within the organism, we back traced the cells that fall under each phenotypic category to the genotype of the mice, meaning male or female mice that express either SP-A1 or SP-A2 (both old and young) or both (young only) as well as SP-A KO mice as control. In order to compare the differences among the phenotypic subpopulation frequencies within the different genotypes, we performed a hierarchical analysis that accounted for effects that may stem from cells originating from the same animal and/or cells that were cultured within the same well. The distribution of the phenotypes for the old mice can be seen in Fig. 5a. There is a significant difference between the male and female SP-A KO mice, with more cells from the male mice seemingly being in a more activated state (compare sums of the “C” and “D” phenotypes between KOM and KOF in Fig. 5a). SP-A2 induces inactivation of macrophages in male mice with an increase of the “A” phenotype. However, SP-A2 has the opposite effect on female mice, as the proportion of cells of the “C” phenotype is increased, at the expense of “A” and “B” cells. This opposite effect of SP-A2 on male and female mice leads to sex differences between the SP-A2 male and female mice. SP-A1 has a similar, albeit more moderate, effect on male mice as SP-A2, i.e., it increases the percentage of cells that are seemingly less active. Similarly to male mice, the effect of SP-A1 on the macrophages from female mice is more moderate than that of SP-A2. Cells of the “C” phenotype are significantly increased in comparison to KOF (*P* = 0.02799), and that effect generates sex differences in the SP-A1 mice as well. Of note, there are no differences between the two variants in either male or female mice.

As far as the young mice are concerned (Fig. 5b), there is a baseline difference between the KO male and female mice, and this trend is opposite to the one in old mice. In the old KO mice, alveolar macrophages with high F-actin content (phenotypes “C” and “D”) are the prevalent phenotypes among males and less than 50 % among females. In the young KO male mice, phenotypes “C” and “D” combined account for ~42 % of the total number of macrophages. The same combination (“C” and “D”) for female mice is 65 %. SP-A2 (but not SP-A1) has an effect on male mice, while in female mice, each variant changes the distribution of AMs significantly. In addition, a significant difference is observed between SP-A1 and SP-A2 in female mice. As is the case with the old mice, there are no differences between the variants in male mice. When both SP-A1 and SP-A2 are expressed, the mice are similar to their KO counterparts, regardless of sex. Interestingly, there are still sex differences in the presence of either one or both variants. In the case of SP-A2, and the combination of both SP-A1 and SP-A2, the sex differences can be attributed to both the “A” and “D” phenotypes, whereas for SP-A1, only cells of the “A” phenotype are significantly different. No differences are observed among male mice that carry at least one SP-A variant, although the SP-A1 vs. SP-A2 *P* value is close to the level of significance, 0.05475. In females, the difference between the two variants shows statistical significance. At the same time, the distribution of cells from SP-A2 mice is similar to the one observed in SP-A1/SP-A2-expressing mice, meaning that the effect of SP-A2 is the major factor driving the observed phenotype.

## Discussion

In a series of studies, we have examined the effects of SP-A on the alveolar macrophage phenotype, as expressed by its cellular proteome, both in vitro and in vivo [14–17]. These studies revealed that the protein expression pattern of the alveolar macrophages is highly dependent on the microenvironment of the cells and the variant of SP-A involved is a major factor affecting the proteome. It was also demonstrated that there are significant sex differences in the response of alveolar macrophages after in vivo treatment of SP-A KO mice with SP-A from human bronchoalveolar lavage [15] or SP-A variants expressed in cell culture [17].

The studies mentioned above showed that the expression of proteins related with the actin cytoskeleton is affected by SP-A. Such proteins include, for instance, the F-actin capping protein, capping protein of the actin filament, the light chain of myosin, and the Rho GDP dissociation inhibitor, among others. Actin-related proteins are clearly of the utmost importance for the alveolar macrophages because many macrophage functions, such as motility, chemotaxis, and phagocytosis, are based on an intact cytoskeletal network with the potential for rapid remodeling.

The present study builds and expands on previous findings regarding the effects of SP-A on the actin cytoskeleton. Using an imaging approach, we determined the effect that SP-A1 and SP-A2 have on the alveolar macrophage phenotype, by studying the distribution of F-actin in the cells. In vitro assays with SP-A variants expressed by CHO cells and use of alveolar macrophages from humanized transgenic mice expressing SP-A1, SP-A2, or both revealed that (i) SP-A1 and SP-A2 differentially affect the alveolar macrophage subpopulations, (ii) the response to SP-A variants differs between males and females, and (iii) the response differs between young and old mice.

Initially, as a proof of concept, we used epifluorescence microscopy to determine whether there are any differences among the alveolar macrophages from mice of different genotypes and different ages, both male and female. It became evident that the phenotype and activation status of the alveolar macrophages, as demonstrated by the cell size and the F-actin mean per pixel fluorescence, is associated with the genotype of the donor animals. The results showed complicated response patterns, especially when factoring in the sex of the animals (Fig. 1). An important observation of this particular experiment was the diversity of phenotypes in the bronchoalveolar lavage cells under baseline conditions, even within the same field of view during imaging. Phalloidin staining of macrophages has been performed before, and the increase in cell size and/or the appearance of filopodia is indicative of macrophage activation, e.g., after LPS challenge [26, 27], while the polymerized actin in the unstimulated macrophages is perinuclear or cortical [28]. It has been reported that M1-activated (pro-inflammatory) macrophages have a dense static actin network (similar to our observed phenotype “B”) whereas the actin of M2-activated (anti-inflammatory) macrophages is more diffuse and randomly distributed (similar to our observed phenotype “C”) [29]. In a study that examined the effects of SP-A on the actin distribution of alveolar macrophages, it was reported that SP-A causes directional expansion of filopodia [30]. Our confocal imaging experiments revealed distinct phenotypes that ranged from actin puncta (phenotype A), dense actin network (phenotype B), diffuse actin network (phenotype C), or protruding filopodia (phenotype D). We consider the distinct phenotypes as representing different stages of activation.

In order to understand whether the SP-A-induced phenotypic changes of the alveolar macrophages are related to rapid cytoskeletal remodeling or more permanent changes, we co-stained cells with fluorescent DNase I, which has been shown to bind to G-actin (monomeric) in the cytoplasm [25]. Comparison of means of G-actin fluorescence units among the phenotypes revealed that the actin cytoplasmic pools follow a trend similar to the one of F-actin (F-/G-actin ratio is not significantly different among the observed phenotypes), indicating that the different phenotypes do not come as a result of rearrangement of the cytoskeleton but probably due to differences in the gene expression of actin itself as well as actin-related proteins. Thus, phenotype “A” is likely to represent cells that are detaching, presumably due to apoptosis, since the actin metabolism in these cells appears to be highly dynamic. Phenotype “B” is probably quiescent, whereas phenotypes “C” and “D” could represent early and late activation status, respectively.

In vitro administration of SP-A1 and SP-A2 proteins from the two SP-A genes to macrophages from SP-A KO mice altered the frequency of each phenotype. SP-A2 caused depolymerization of F-actin in cells from females, as demonstrated by the increase of phenotype A cells. When both SP-A1 and SP-A2 were used to treat AMs from KO mice, moderate effects were observed in both sexes compared to SP-A2 alone. This can be explained by the potential counterbalancing actions of SP-A1 and SP-A2. The higher proportion of activated alveolar macrophages from male mice exposed to SP-A2 (Fig. 4) is in accordance with a functional assay previously reported by our lab [13]. In that study, alveolar macrophages from male rats were challenged with *P. aeruginosa* in the presence or absence of SP-A1 or SP-A2, and it was found that the phagocytic index of cells exposed to SP-A2 was higher than that of cells exposed to SP-A1.

The opposing actions of SP-A1 and SP-A2 are supported by the ex vivo results from the present study (Fig. 5). Cells isolated from young humanized transgenic male mice carrying SP-A1 did not show a significantly different phenotype distribution compared to KO, but cells from SP-A2 males showed a higher proportion of cells of the “C” and “D” phenotypes compared to KO, meaning that the macrophages of these mice are readily active. These results are consistent with our previous work [17] which showed that the proteome of macrophages from male mice is not as responsive to SP-A administration in vivo as females, as far as the actin-related group of proteins is concerned. Indeed, the present study verifies that in female mice, there is significant response to both SP-A1 and SP-A2 and this response is different between the two variants. However, co-expression of both variants counterbalances the effect and brings the cells to a distribution similar to the one observed in KO mice. Even though the distribution pattern is similar between the KO and the co-expressing cells, we speculate that these cells may be functionally distinct, with the cells exposed to SP-A1 and SP-A2 being primed for activation, whereas the KO cells may not. Further studies are needed to elucidate this.

Unlike the young male mice, cells isolated from older male transgenic mice were different from the KO. The punctate pattern of F-actin was more prominent in both SP-A1 and SP-A2 mice, with SP-A2 showing a higher proportion of cells with low actin levels. In older female mice, there are also gene-specific differences, with SP-A2 demonstrating more cells of the “C” phenotype and SP-A1 having higher proportion of the “D” phenotype. Aging has been reported to affect the immune system in general and macrophage functions in particular. Impairments of the immune system that are related to age (termed immunosenescence) seem to contribute to increased susceptibility to infectious diseases, as well as cancer and autoimmunity [31]. In splenic macrophages, TLR4 signaling has been shown to be compromised during aging which leads to a perturbed pattern of cytokine expression [32, 33], although the exact molecular mechanisms are not well understood. Importantly, SP-A has been shown to directly interact with TLR4 [34] and its co-receptor CD14 [35] and modulates the TLR4 activity. Reduced expression of CD14 has been proposed as the reason for the impaired TLR4-related signaling during aging [32]. Macrophage polarization towards the M1 and M2 phenotypes has also been reported to be affected by age. Although age does not result in a skew towards either phenotype [36], old mice seem to have higher numbers of M2 macrophages in the spleen, lymph nodes, and bone marrow [37]. Interestingly, this observation could be in accordance with our study, if we consider alveolar macrophages of the “C” phenotype in the present study to be similar to M2 polarized, as described elsewhere [29, 38].

There are sex differences in the distribution of subpopulations of AMs among mice of the same genotype in both the in vitro (Fig. 4) and the ex vivo (Fig. 5) experiments. These results come as a continuation of a long series of studies that have demonstrated such differences in both the molecular and the physiological level [3, 4, 15, 17, 39, 40]. Hormonal regulation of the actin cytoskeleton in AMs could be an important factor in the generation of sex differences. Sex hormones have been shown to affect cytoskeletal proteins in other systems [41–43] and also the production of surfactant in the developing lung [44–46]. Whether sex hormones actually affect the cytoskeleton of AMs through SP-A or other mechanisms remains to be investigated.

Functionally, the regulation of the distribution of subpopulations of AMs by SP-A variants may explain differences observed in their phagocytic activity [11–13] and the course of lung disease [4, 39, 40, 47]. Sex differences concerning susceptibility in lung disease, such as asthma [48], chronic obstructive pulmonary disorder [49], and even lung cancer [50], have been widely reported, although the latter remains controversial [51]. A more detailed analysis of the mechanisms governing the regulation of AM function by SP-A could provide clues to the development of individualized treatment according to a person’s genotype, as the AMs are the first line of defense. Such a treatment targets directly the AMs, and this could eliminate or minimize the need for intervention to activate the adaptive immune response, which would be even more complicated and perhaps more harmful to the host.

## Conclusions

We examined the effects of surfactant proteins A1 and A2 on the alveolar macrophage phenotypes of male and female mice. Our results demonstrate that there are subpopulations of AMs as demonstrated by the actin cytoskeleton staining of the cells. Using humanized transgenic mice as a model, we showed that the exposure of AMs to SP-A1 or SP-A2 results in differential effects on macrophage phenotypes. These effects are ablated by exposure to both proteins, suggesting that these proteins exert opposing effects on the macrophages. Moreover, the phenotype responses of cells from male and female mice showed different responses to these proteins, a fact that should be examined more closely in the view of surfactant treatments. The age of the mice is also a major factor as it plays a role in the response of AMs to SP-A variant exposure. This indicates the importance of the SP-A genotype at the level of immune response compromises during aging. Our findings demonstrate the importance of SP-A in the immune response of the lung, but also illustrate the need for consideration of sex and age in studies that investigate lung immune responses and pathology.

## Abbreviations

BAL: bronchoalveolar lavage
F-actin: filamentous actin
G-actin: globular actin
KO: knockout
LPS: lipopolysaccharide
mAMs: (mouse) alveolar macrophage(s)
SP-A: surfactant protein A
SFTPA1: surfactant protein A1 gene
SFTPA2: surfactant protein A2 gene
